# Intracellular Vesicle Acidification Promotes Maturation of Infectious Poliovirus Particles

**DOI:** 10.1371/journal.ppat.1003046

**Published:** 2012-11-29

**Authors:** Alexsia L. Richards, William T. Jackson

**Affiliations:** Department of Microbiology and Molecular Genetics, Medical College of Wisconsin, Milwaukee, Wisconsin, United States of America; University of Southern California School of Medicine, United States of America

## Abstract

The autophagic pathway acts as part of the immune response against a variety of pathogens. However, several pathogens subvert autophagic signaling to promote their own replication. In many cases it has been demonstrated that these pathogens inhibit or delay the degradative aspect of autophagy. Here, using poliovirus as a model virus, we report for the first time *bona fide* autophagic degradation occurring during infection with a virus whose replication is promoted by autophagy. We found that this degradation is not required to promote poliovirus replication. However, vesicular acidification, which in the case of autophagy precedes delivery of cargo to lysosomes, is required for normal levels of virus production. We show that blocking autophagosome formation inhibits viral RNA synthesis and subsequent steps in the virus cycle, while inhibiting vesicle acidification only inhibits the final maturation cleavage of virus particles. We suggest that particle assembly, genome encapsidation, and virion maturation may occur in a cellular compartment, and we propose the acidic mature autophagosome as a candidate vesicle. We discuss the implications of our findings in understanding the late stages of poliovirus replication, including the formation and maturation of virions and egress of infectious virus from cells.

## Author Summary

The autophagic degradation pathway is a well-known agent of innate immunity. Several pathogens, including poliovirus (PV), a model for several medically important RNA viruses, subvert this pathway for their own benefit. In doing so, pathogens often inhibit the degradative portion of the pathway, presumably to prevent their own destruction. We show here that, surprisingly, PV infection results in high levels of degradative autophagy. However, we find that autophagic degradation is dispensable for PV replication. Inhibiting the formation of autophagosomes inhibits virus RNA replication and subsequent steps in virus production. Inhibiting the acidification of vesicles, which in the case of autophagosomes precedes fusion with lysosomes and autophagic degradation, inhibits a much later step in virus production. Our data suggest an important role for an acidic compartment of the cell in the final maturation step, cleaving a capsid protein to generate infectious virus. Importantly, these data also call into question the long-standing hypothesis that all steps in the production of infectious poliovirus are cytosolic.

## Introduction

The *Picornaviridae*, a family of non-enveloped viruses with a small positive strand RNA genome, includes numerous known and emerging pathogens of medical, veterinary and agricultural importance [1]. Poliovirus (PV) is the most extensively studied virus in this family in terms of our collective understanding of its molecular and cellular biology, biochemistry, structure, life cycle, and pathogenesis and is therefore an important model system. Infection with PV results in numerous changes to the host cell, and perhaps one of the most notable is the massive accumulation of cytosolic double-membraned vesicles [2], [3]. These vesicles are the hallmark of autophagy, a degradative pathway of homeostasis and stress response [4].

Autophagy begins with generation of novel membrane crescents which, as they expand and self-fuse, sequester cytoplasmic contents in double-membraned vesicles, referred to as autophagosomes [5]. As autophagy is induced, increasing amounts of the cellular autophagy protein LC3 become conjugated to the lipid phosphatidylethanolamine. This conjugation confers membrane association and is required for autophagosome formation and membrane expansion [6], [7]. Autophagosomes fuse with endosomes to form amphisomes. This fusion event provides amphisomes with vacuolar ATPases, and results in their acidification [8], [9]. Subsequent to acidification, amphisomes fuse with lysosomes to form single-membraned autolysosomes and cargo is degraded [9]–[11].

Poliovirus specifically induces autophagic signaling, and virus production correlates with the level of autophagic activity in cells. LC3 lipidation is evident as early as 3 hours post-infection (h.p.i.), and by 5 h.p.i. double-membraned vesicles are found throughout the cytoplasm [12], [13]. These vesicles are positive for both LC3 and the endosomal marker LAMP1, indicating that these vesicles have likely fused with late endosomes. PV-induced vesicles also stain with monodansylcadaverine (MDC), a lysosomotropic agent is concentrated in acidic compartments by an ion-trapping mechanism [12], [14]. MDC staining co-localizes with LC3 and LAMP1, indicating that the induced vesicles are likely to be acidic amphisomes. Components of the PV replication complex are located on these vesicles, leading to speculation that they may be the sites of genome replication [12], [15].

Although autophagic signaling is specifically induced by the action of PV proteins, the fate of the induced autophagosomes has not been investigated [12], [13], [15]. The regulation of autophagy can be broadly placed into two classes. One class controls the initiation of autophagosome formation by regulating mamalian target of rapamycin (mTOR) inhibition and subsequent LC3 lipid conjugation [16]. The second class controls the stepwise maturation of autophagosomes into degradative autolysosomes. Inhibitors of vesicle acidification, including the weak base ammonium chloride and the vacuolar ATPase inhibitor bafilomycin A1, have been shown to inhibit amphisome fusion with the lysosome and subsequent degradation of cargo [17]–[19].

The presence of late regulatory mechanisms mean that the formation of autophagosomes can occur without leading to active degradation. During infection with the picornavirus Coxsackievirus B3 (CVB3) double-membraned autophagosomes are observed, but autophagic degradation does not occur [20], [21]. Similarly, the bacteria *Legionella pneumophila* induces autophagosomes for use as replicative vesicles, but the bacterium secretes factors that delay maturation and fusion with lysosomes [22]–[24]. By inhibiting the degradative portion of the pathway, these pathogens are thought to maximize the benefits of autophagosome formation.

The production of the flavivirus Dengue Virus 2 also correlates with the level of autophagic activity in the cell. Unlike the previous examples, Dengue virus does not appear to replicate its RNA on or within autophagosomes [25]. A series of elegant experiments demonstrated that the virus benefits from the selective autophagic degradation of lipid droplets, known as lipophagy [26]. When lipophagy is inhibited, virus production is reduced. This effect is reversed when cells are supplemented with the products of lipophagy.

These data highlight the remarkable diversity in the ways that viruses subvert the autophagic pathway, and raise the possibility that autophagic degradation could itself promote virus production. Inhibitors of vesicle acidification, which would be expected to inhibit autophagic degradation, have been shown to inhibit infection with several viruses including Semliki Forest virus and human rhinovirus 2 [27], [28]. However, these effects are thought to be primarily associated with elevated pH of the endocytic entry vesicles and not related to autophagy. Previous studies have shown that PV entry, translation, and polyprotein processing are unaffected by these inhibitors [29]. These studies did not investigate overall infectious virus production.

Here we show that PV induces *bona fide* autophagic degradation, although the degradation is not required for normal virus production. We go on to show that formation of autophagosomes promotes viral RNA replication while acidification of cellular vesicles promotes a post-RNA replication step of infectious virus production. Specifically, we find that maturation of assembled particles into infectious virions is promoted by acidic compartments. We suggest that particles which assemble within, or those captured by, autophagosome-like vesicles are exposed to a low-pH environment, facilitating maturation of infectious virus.

## Materials and Methods

### Viruses, cells, and plaque assays

Poliovirus Mahoney type 1 was isolated following transfection with an infectious cDNA [30] and propagated as previously described [31]. Poliovirus stocks were titered on H1-Hela cells. H1-Hela cells were maintained in MEM+10% calf serum (CS). 293T cells were maintained in DMEM+10% fetal bovine serum. For collection of intracellular virus cells were washed with PBS, then collected in 1 mL PBS+ 100 µg/mL MgCl_2_ and 100 µg/mL CaCl_2_. Cells were lysed by three cycles of freeze/thawing. Virus was added to monolayers of H1-Hela cells for a 30 minute absorption, after which cells were overlaid with 1% agar in MEM. Plaques were allowed to develop for 48 h, agar overlay was removed and cells stained with crystal violet.

### Reagents (Antibodies and chemicals, RNA interference)

MG132, ammonium chloride (NH_4_Cl), E64d, pepstatin A, 3-methyladenine (3-MA), and guanidine HCl were purchased from Sigma. Bafilomycin A1 and a polyclonal antibody against p62 were obtained from Santa Cruz Biotechnology. Polyclonal antibodies against GAPDH and LC3 were purchased from Cell Signaling and MBL respectively. LC3A and LC3B as well as irrelevant siRNA was purchased from Dharmacon and were transfected using Lipofectamine 2000 (Invitrogen) according to the manufacturers' instructions.

### Quantitative Real-Time PCR

Total RNA was harvested from infected cells using Trizol (Invitrogen) according to the manufacturers instructions. Four micrograms of RNA were treated with DNA*-free* DNase treatment (Ambion) and split into two reactions. One half was subjected to reverse transcription using RevertAid First Strand cDNA Synthesis Kit (Fermentas) and oligo-dT primers. The second half of DNase treated RNA was subjected to mock reverse transcription in the absence of the enzyme (“No RT” control). cDNA was serially diluted (8-fold), including “No RT” controls, and measured, in triplicate for each dilution, by real time PCR using iCycler (Bio-Rad). Virus-specific primers were designed by PrimerQuestSM (Integrated DNA Technologies) and used to amplify cDNA. Primers were to the PV genome (TATGATGCATCTAGCCCTGCT and ACAGGTGGTGTGAGTGGT TTAGGT) and GAPDH (TGTGATGGGTGTGAACCACGAGAA and GAGCCCTTCCACAATGCCAAAGTT). The delta-Ct method was used to quantify relative abundance of viral cDNA. Ct values of “No RT” controls did not exceed background levels.

### Western blots

Cells were harvested with phosphate-buffered saline (PBS) containing 55 mM EDTA (pH 8.0) and collected by centrifugation at 1500 rpm for 5 min. Cell pellets were washed in PBS and pelleted by centrifugation at 7500 rpm. Cell pellets were resuspended in RSB-NP-40 (10 mM Tris-HCl [pH 7.5], 10 mM NaCl, 1.5 mM MgCl_2_, 1% NP-40) supplemented with Mini-complete EDTA-free protease inhibitor cocktail (Roche Applied Science, Indianapolis, IN), and incubated on ice for 15 min. Nuclei and insoluble debris were pelleted by centrifugation at 7500 rpm for 5 min. Cell extracts were then stored at −20°C or subjected immediately to sodium dodecyl sulfate-polyacrylamide gel electrophoresis (SDS-PAGE). Immediately following separation proteins were transferred to PVDF membranes (Thermo Scientific). Membranes were blocked for 1 h with 5% nonfat dry milk solution in Tris-buffered saline containing 1.0% Tween 20 (TBST). Blots were then incubated with the primary antibody, washed with TBST, and incubated for 1 hr with secondary antibody. Immunoreactive bands were visualized by enhanced chemiluminescence (HyBlot Cl, Deville Scientific).

### Protein labeling

Cells were infected at an MOI of 50 pfu/cell. Protein labeling was performed with 20 µCi of [^35^S] methionine per mL in methionine-free medium. The radiolabeled cell monolayers were collected in 1 mL PBS and centrifuged at 4500 rpm for 5 minutes. Cells were then resuspended in 30 µL RSB-NP-40 (10 mM Tris-HCl [pH 7.5], 10 mM NaCl, 1.5 mM MgCl_2_, 1% NP-40) supplemented with Mini-complete EDTA-free protease inhibitor cocktail (Roche Applied Science, Indianapolis, IN), and incubated on ice for 15 min. Nuclei and insoluble debris were pelleted by centrifugation at 7500 rpm for 5 min. Radiolabeled cell lysates were then stored at −20°C. Proteins were separated on a 13% polyacrylamide gel. Images obtained with the Typhoon FLA 9500 (GE Healthcare Life Sciences) and ImageQuant software (Amersham Biosciences).

### Analysis of assembly intermediates by sucrose gradients

After 3 h of incubation, the cells were washed twice with DME lacking methionine (GIBCO), 3.0 ml of DME lacking methionine, containing 100 µCi of [^35^S]methionine (ICN) per ml was added to each plate. Cells were incubated for either 2 or 3 hours at 37.0°C. Cells were washed and harvested by scraping into 1 ml of a solution containing 10 mM Tris (pH 7.4), 10 mM NaCl, 1.5 mM MgCl_2_, 1% Nonidet P-40, 1 uM phenylmethylsulfonyl fluoride (Sigma), and 40 U of placental RNasin (Promega) per ml. Nuclei were pelleted by centrifugation at 1,600×g for 10 min at 4°C, and 0.5 ml of the resulting cytoplasmic extract was loaded directly on 11-ml gradients containing 15 to 30% sucrose in 10 mM Tris (pH 7.4)-10 mM NaCl-1.5 mM MgCl_2_. The particles were sedimented through the sucrose gradients by centrifugation for 3 h at 27,500 rpm at 15°C, with a Beckman SW41 rotor. Viral proteins were separated on a 15% polyacrylamide gel. Images obtained with the Typhoon FLA 9500 (GE Healthcare Life Sciences) and ImageQuant software (Amersham Biosciences).

## Results

### p62 degradation following PV infection demonstrates active autolysosome degradation

**Figure 1:**
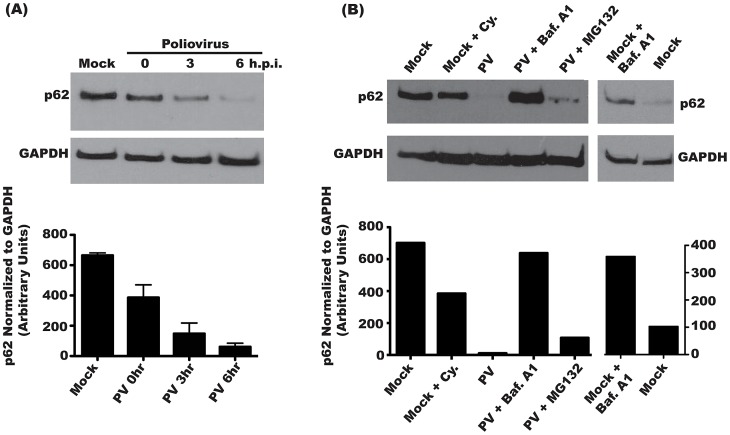
Steady state levels of p62 decrease during poliovirus infection. (A) H1-Hela cells were mock infected or infected with poliovirus (MOI = 50 pfu/cell) and cells were lysed at the indicated hours post-infection (h.p.i.). Three independent experiments were measured for quantification of p62 levels, and the blot shown is representative. (B) H1-Hela cells were mock infected or infected with poliovirus (MOI = 50 pfu/cell) and cells were lysed at 6 h.p.i. Cycloheximide (Cy.) was added to mock infected cells at a final concentration of 50 µg/mL, for 4 hours. Cells were then pre-treated with 0.1 µM bafilomycin A1 (Baf.A1) for 14 hours prior to infection and kept under treatment throughout infection. MG132 was used at a final concentration of 20 µM and added to the media at the time of infection. The blot shown is representative of three independent experiments.

Poliovirus induces autophagic signaling during infection [12], [32]. Several other pathogens induce autophagic signaling during infection, but subsequently inhibit the degradation of autophagic cargo [21], [33]–[35]. To ask if poliovirus infection results in autophagic degradation, we monitored the steady-state level of a cellular protein, p62, over the course of infection. p62 is incorporated into autophagosomes though direct interaction with LC3 on the autophagosome membrane [36]. p62 is degraded following fusion of the autophagosome with the lysosome; therefore, a decrease in cellular levels of p62 reflects increased autophagic degradation [37]. We found that p62 levels begin to decrease by 3 h.p.i. The signal continues to decline over the course of infection, with over 90% of p62 signal gone by 6 h.p.i. (**Figure 1A**) Since poliovirus inhibits cellular translation, we considered that the loss of p62 signal could be the result of normal protein turnover. To test this, mock-infected cells were incubated with cycloheximide for 4 hours to inhibit cellular translation. We observed approximately 47% reduction in p62 levels, likely due to background autophagy, which is high in HeLa cells [38]. However, this background autophagy is insufficient to account for the p62 reduction observed during poliovirus infection. To further elucidate the mechanism of poliovirus-induced p62 degradation, infections were performed following inhibition of either the ubiquitin-proteasome or the lysosomal degradation pathway. Treatment with MG132, a specific inhibitor of the 26S proteasome, resulted in only a 13% rescue of p62 degradation following PV infection. Treatment of cells with bafilomycin A1, an inhibitor of vacuolar ATPases, inhibits fusion of amphisomes with lysosomes [18]. In the presence of bafilomycin A1, PV infection did not reduce p62 levels (**Figure 1B**).

**Figure 2:**
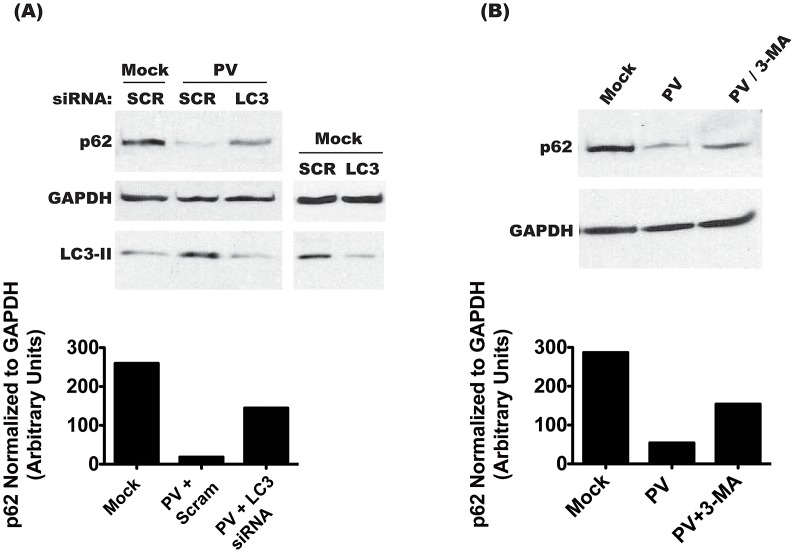
Degradation of p62 is dependent on the induction of autophagy. (A) Cells were transfected with scrambled (SCR) or anti-LC3 (LC3) siRNAs, and 48 h later infected with PV at an MOI of 50 pfu/cell, or mock infected. Cells were collected at 6 h.p.i., and immunoblots were performed on lysates for p62, GAPDH, and LC3. (B) Cells were treated with 10 mM 3-MA for 12 h, then infected with PV at an MOI of 50 pfu/cell, or mock infected. Cells were collected at 6 h.p.i. and lysates were used for immunoblots.

To determine if p62 degradation is specific to autophagy, we transfected cells with siRNA specific to LC3A and LC3B, the predominant splice variants of LC3 used in cellular autophagy (**Figure 2A**). As a control, we transfected with scrambled, irrelevant siRNA. We achieved a 78% knockdown of LC3 protein, as estimated by Western blot in mock-infected cells. In PV-infected cells, the levels of membrane-bound LC3-II increase due to induction of autophagic signaling [32]. LC3 siRNA treatment reduced LC3-II to levels comparable to that of uninfected cells, likely due to a reduction in the overall amount of LC3 available for modification. This level of knockdown resulted in restoration of 55% of wild-type p62 during infection. To confirm these results, we used the autophagy inhibitor 3-methyladenine (3-MA), which reduces autophagy signaling by inhibiting type-III PI-3 kinase activity [39]. As seen in **Figure 2B**, treatment with 3-MA restored over 50% of p62 levels during infection. These data show that PV, which benefits from autophagosome formation, also allows active autophagic degradation during infection.

### Lysosomal protease inhibitors do not affect yield of intracellular poliovirus

**Figure 3:**
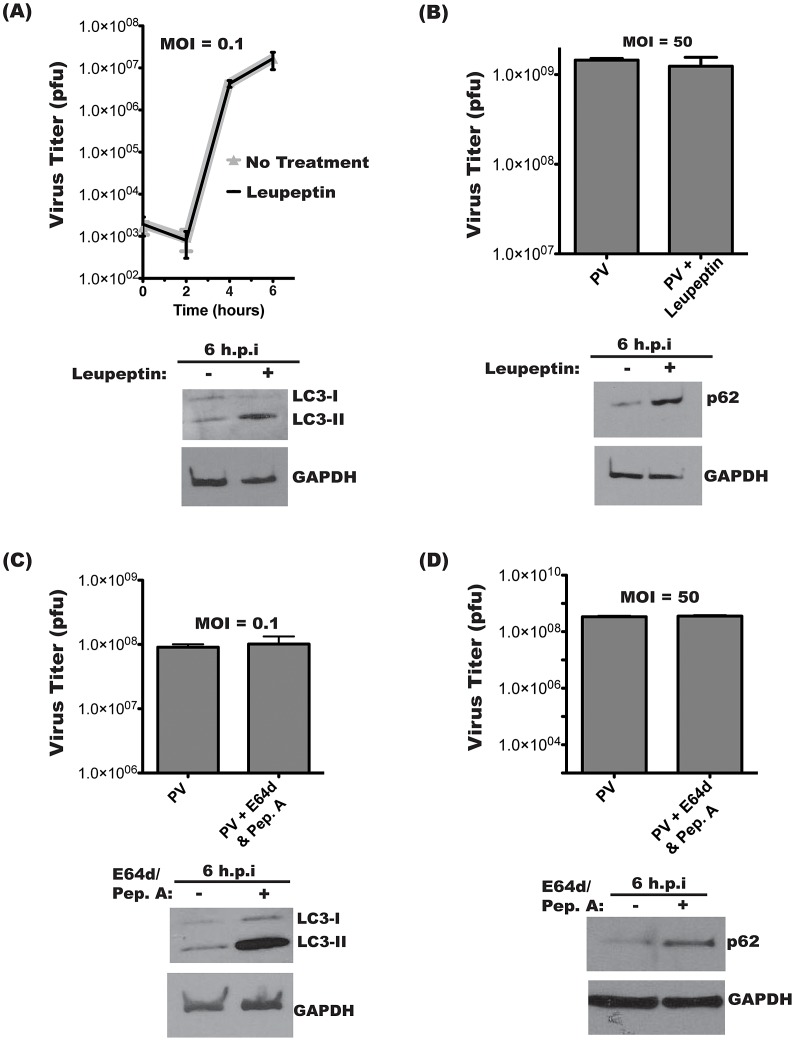
Intracellular poliovirus yields are not affected by lysosomal protease inhibitors. Triplicate samples of H1-Hela cells were infected with PV at an MOI of 0.1 pfu/cell (**A**) or MOI of 50 pfu/cell (**B**). Cells were pre-treated with 20 µM leupeptin for 14 hours prior to infection and kept under treatment throughout infection. Cell-associated virus was collected at the indicated times post-infection, and virus titers were determined by plaque assay. Parallel infections were collected for Western blots of LC3 (A) or p62 (B) In (B) cell-associated virus was collected at 6 h.p.i. for plaque assay. (**C**) Infections were performed as in (A), then 10 µg/mL each of E64d and Pepstatin A (Pep.A) were added to the media at the time of infection. Inhibition of lysosomal degradation was confirmed by immunoblot for LC3 and GAPDH. (**D**) Infection at an MOI of 50 pfu/cell, with E64/Pep.A as in (C).

We next wanted to investigate if the virus benefits from active autophagic degradation. PV infections were performed at low (**Figure 3A, C**) or high (**Figure 3B, D**) multiplicity of infection (MOI) in the presence of lysosomal protease inhibitors. No effect on virus yield was observed when cells were pre-treated with leupeptin, a thiol protease inhibitor, and infected at an MOI of 0.1 pfu/cell [40]. To ensure that leupeptin was inhibiting autophagic degradation, lysates from parallel samples were probed for the autophagosome marker LC3. (**Figure 3A**). Lipidated LC3 (LC3-II) is incorporated into the autophagosome membrane and is specifically degraded by the autolysosome [**41]**–[43]. A decrease in background autophagic turnover results in increased LC3-II levels, so, as expected, cells treated with leupeptin showed an increase in LC3-II [38].

We wanted to test the effect of inhibiting autophagic degradation at a high MOI, so, as shown in **Figure 3B**, we repeated the leupeptin experiment at an MOI of 50 pfu/cell. For high MOI infections, it is possible to directly assay autophagic degradation by turnover of p62. Our Western blot demonstrates inhibition of PV-induced p62 degradation. To confirm that these results were not limited to leupeptin treatment, infections were performed in the presence of a combination of protease inhibitors E64d, a calpain and cathepsin B inhibitor, and pepstatin A, an inhibitor of aspartic proteases [40], [44]. As shown in **Figure 3C**, cells were infected at MOI of 0.1 pfu/cell and treated with both E64d and pepstatin A, and no difference in virus yield was observed. LC3-II levels significantly increased following treatment with the protease inhibitors, indicating that lysosomal degradation had effectively been restricted. **Figure 3D** demonstrates treatment with E64d and pepstatin A during high MOI infections, with no effect of viral yield. As in Figure 3B, a high MOI infection allows us to assay p62 levels. As expected, E64d/Pep.A abrogates PV-induced degradation of p62. These results show that inhibiting lysosomal degradation does not inhibit virus replication regardless of the nature of the inhibitors or the MOI of the infection.

### Inhibitors of vesicle acidification reduce yield of intracellular poliovirus

**Figure 4:**
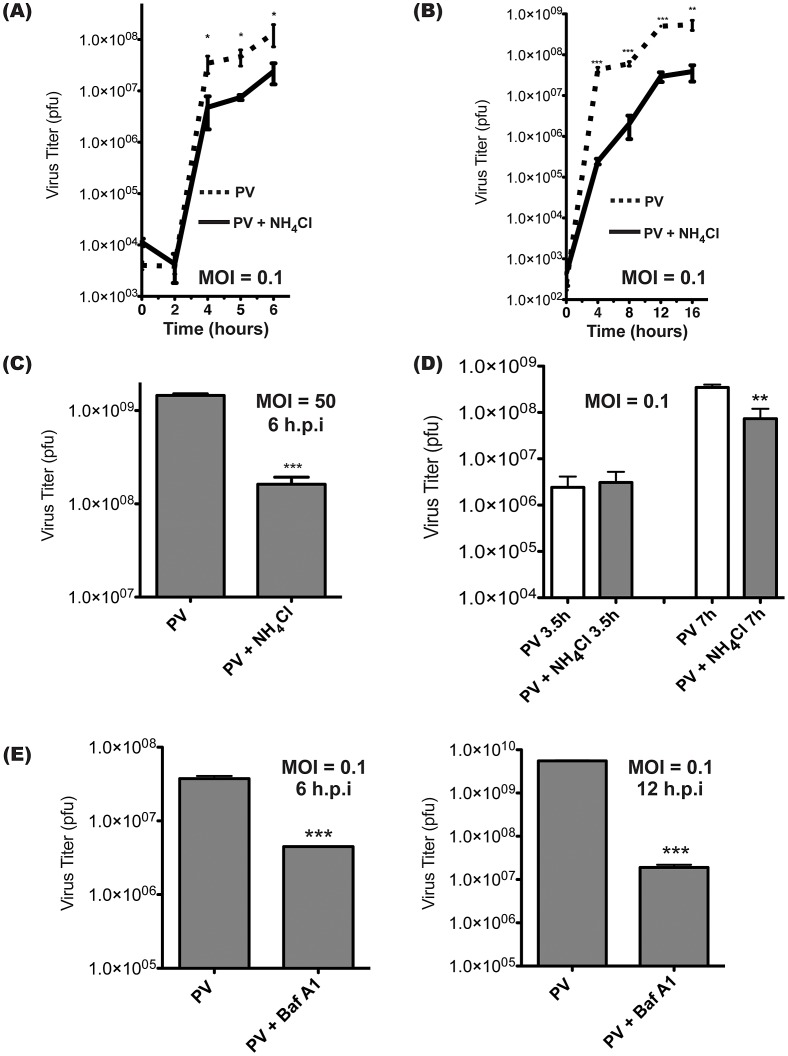
Intracellular poliovirus yields are reduced when cells are treated with inhibitors of vesicle acidification. (A) Triplicate samples of H1-Hela cells were infected with PV at an MOI of 0.1 pfu/cell, and cell-associated virus was collected at the indicated times post-infection. NH_4_Cl (20 mM) was added to the media at the time of infection (solid line). Virus titers were then determined by plaque assay. (B) Infection as in (A), carried out to 16 h to represent a multiple-cycle infection. (C) Triplicate H1-Hela infections with PV at an MOI of 50 pfu/cell, with cell-associated virus collected at 6 h.p.i. (D) Cells were infected as in (A), then NH_4_Cl was added to half of the samples at 3.5 hours post-infection, and cell-associated virus was collected immediately or at 7 h.p.i. (E) Cells were pre-treated for 14 hours with 0.1 µM bafilomycin A1 (Baf.A1) and kept under treatment throughout infection (MOI = 0.1 pfu/cell). Cell-associated virus was collected at 6 or 12 h.p.i. and virus titers were determined by plaque assay. * p<0.05, ** p<0.01, *** p<0.0001.

The lumen of the amphisome reaches an acidic pH of approximately 5.7 [9]. To investigate whether this acidification is important for virus production, cells were treated with inhibitors of vesicle acidification during infection. Ammonium chloride (NH_4_Cl) is a weak base that is taken up by intracellular vesicles [45]. The acidic environment results in protonation of the base, which raises the lumenal pH as it diffuses out of the vesicle [46]. Cells were infected with PV, then treated with NH_4_Cl immediately after absorption of the virus. NH_4_Cl treatment reduced the production of infectious virus progeny by approximately one log at later time points. (**Figure 4A**) Since these are low MOI infections, we wanted to determine if the effect of NH_4_Cl treatment is due to a delay of the infectious cycle or an overall reduction in PV production. In **Figure 4B**, we carry the infection out to 16 hours and find that the reduction of PV production in the presence of NH_4_Cl is maintained throughout.

We next wanted to determine if the effect we were seeing was specific to low MOI infections. H1-HeLa cells were infected at an MOI of 50 pfu/cell and cell-associated virus was collected at 6 h.p.i. (**Figure 4C**) The results were strikingly similar to our low MOI infection experiments, with an approximately one-log reduction in viral titer, indicating that the effect is independent of MOI. We also wanted to investigate if the effects of acidification inhibitors would be diminished if they were added following the peaks of both viral translation and transcription. NH_4_Cl was added to cells at 3.5 h.p.i. to limit any possible effects on virus entry or the initiation of virus transcription or translation [47]. Cells were then lysed at 7 h.p.i., and plaque assays revealed an 8-fold reduction of infectious virus production. (**Figure 4D**) These data are similar to the results in Figure 4A, indicating the effect of NH_4_Cl on the virus life cycle primarily takes place later than 3.5 h.p.i.

To ensure that the effect observed was not specific to treatment with NH_4_Cl, cells were pre-treated with bafilomycin A1, a specific inhibitor of the vacuolar ATPase responsible for vesicle acidification [48]. Infection with poliovirus following bafilomycin A1 treatment resulted in a 6-fold reduction in infectious virus at 6 h.p.i. (**Figure 4E**) We carried this time course out to 12 h.p.i., beyond a single viral replication cycle, and found that the effect on viral titer is even more pronounced than in the 6 hour infection, although this may be due to compounding effects that result from the extended pre-treatment with bafilomycin A1 prior to infection. We conclude that inhibiting vesicle acidification reduces infectious poliovirus production significantly.

### Inhibitors of vesicle acidification do not alter viral RNA replication

**Figure 5:**
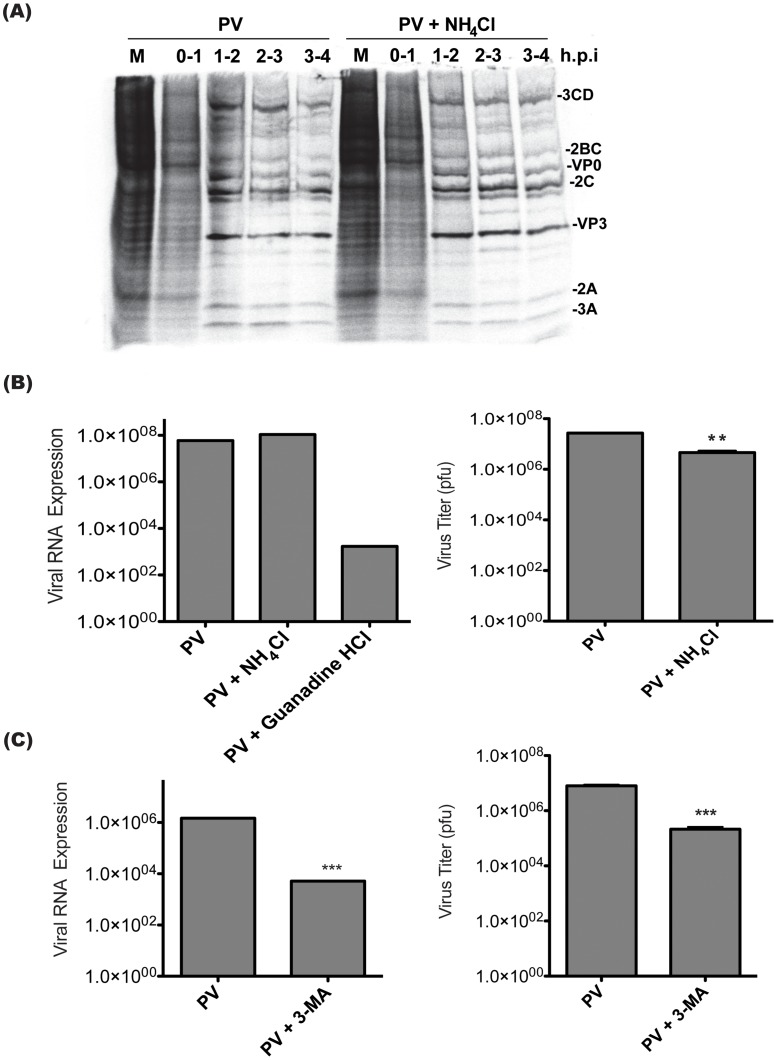
Poliovirus entry, translation, and RNA replication are unaffected by treatment with inhibitors of vesicle acidification. (**A**) H1-HeLa cells were infected with PV at an MOI of 50 pfu/cell. Cells were pulsed at the indicated h.p.i with ^35^S-labeled methionine for 1 h then lysed. Lysates were run on SDS-PAGE. Expected viral proteins are labeled according to recognized banding patterns. (**B**) Triplicate plates of H1-Hela cells were infected with PV at an MOI of 0.1 pfu/cell, virus RNA and host GAPDH RNA were measured by qRT-PCR. Virus RNA levels were normalized to GAPDH levels using the delta-Ct method. NH_4_Cl treatment was as described in Figure 4, and Guanidine HCl (2 mM) was added to the media at the time of infection. The data shown are pooled from three replicate experiments, and the titer of cell-associated virus collected at 6h.p.i. from each replicate was determined by plaque assay. (**C**) Triplicate plates of 293T cells were treated with 20 mM 3-MA for 2 hours prior to infection, and kept under treatment throughout infection. PV infections were done at an MOI of 0.1 pfu/cell. RNA levels and virus titers were analyzed as in (B). ** p<0.01, *** p<0.0001.

We wanted to understand the reason for the decrease in viral yield when vesicle acidification is inhibited. Poliovirus does not require a pH change in the entry vesicle to enter cells and release its genome, nor to translate and process the viral polyprotein [29]. However, we wanted to test this using our inhibitors and protocols to ensure that acidification of the entry vesicle was not affecting these early steps in the virus replication cycle. To do this, we used pulse labeling to detect translation of viral proteins. Because PV efficiently inhibits protein synthesis, ^35^S-Methionine labeling at discrete time points during infection should reveal that viral proteins make up the vast majority of protein production within a few hours of infection [49], [50]. This indicates that virus was able to enter cells, release its genome, and initiate translation. By comparing the pattern of proteins produced to untreated infected cells, we can identify changes in polyprotein processing as well. We observed no significant differences in the pattern of protein labeling from infections done in the absence or presence of NH_4_Cl. (**Figure 5A**) We conclude that virus entry, translation and polyprotein processing are not affected by NH_4_Cl treatment.

To ask if defects in viral RNA replication can explain the decrease in virus yield following NH_4_Cl treatment, qRT-PCR was performed using poliovirus specific primers to detect changes in viral genome replication. To ensure that our assay was able to detect changes in viral RNA levels, infections were also done in the presence of guanidine HCl, a specific inhibitor of poliovirus RNA replication [51]. Treatment with guanidine HCl resulted in significantly decreased viral RNA levels at 6 h.p.i. No change in viral RNA levels was observed at 6 h.p.i. when infections were carried out in the presence of NH_4_Cl. (**Figure 5B**) The reduced titer of intracellular virus from each replicate shows that NH_4_Cl reduces the yield of intracellular virus at 6 h.p.i., despite normal levels of viral genome replication.

Although tools do not exist to specifically inhibit acidification of the autophagic subset of vesicles, we can inhibit their formation using 3-MA. H1-HeLa cells have high levels of autophagy, and it is difficult to achieve significant reduction of autophagic levels. To examine cells in which autophagy can be efficiently inhibited, we turned to the 293T cell line, in which baseline autophagy levels are much lower [52]. (**Figure S1A**) We examined LC3 modification following PV infection and found that 3-MA was significantly inhibiting viral induction of the autophagic pathway in 293T cells but not in H1-HeLa cells. (**Figure S1B**) It is worth noting that although we could consistently detect slightly lower amounts of virus when H1-HeLa cells were infected in the presence of 3-MA, we were unable to identify changes in RNA levels or any other step in virus production. (**Figure S1C–E**) In 293T cells, 3-MA treatment led to a two-log reduction in viral genomic RNA levels at 6 hours post infection and a one-log reduction in virus titer. (**Figure 5C**) Our data indicate that inhibiting autophagosome formation and inhibiting vesicle acidification have different effects on the virus life cycle.

### Maturation of virions is promoted by vesicle acidification

There are, essentially, three events which must occur to produce infectious virus from genomic RNA and capsid proteins. The first, assembly of pentamers from capsid protein, is thought to occur spontaneously [53]. The second step, association of the genomic RNA with pentamers, is thought to nucleate assembly of 150S provirions, named after their sedimentation coefficient on a sucrose gradient [54]. After virions assemble, the final step is a cleavage maturation of one of the capsid proteins, VP0, which is cleaved into VP4 and VP2 resulting in the infectious 150S virion. This is believed to be an autocatalytic reaction and there is no published evidence of its pH-dependence [55], [56]. The specific mechanism of virus particle maturation is unclear.

**Figure 6:**
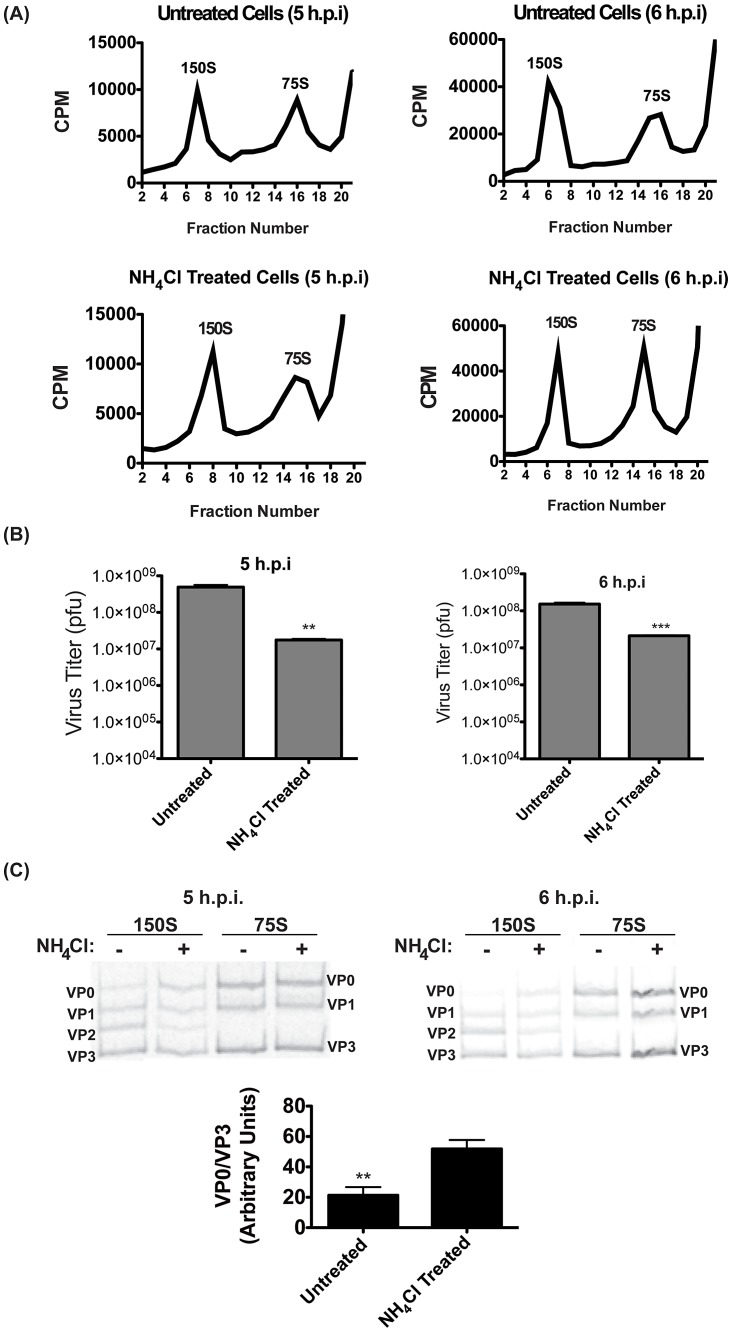
Virion maturation requires acidic compartments. (**A**) H1-Hela cells were infected at an MOI of 50 pfu/cell, and half of the samples were treated with NH_4_Cl. Cells were labeled with ^35^S-Methionine from 3 h.p.i. until collection at 5 or 6 h.p.i., and lysates were then separated on a 15–30% sucrose gradients. Fractions were then collected and the counts per minute (CPM) were analyzed for each fraction. Representative gradients from three independent experiments are shown. (**B**) The three fractions representing the 150S peak in each experiment were pooled for plaque assay analysis. Data shown are the averages from three independent experiments. (**C**) Three fractions representing the 150S and 75S peaks were pooled and run on SDS-PAGE, and the ^35^S-Methionine labeled bands were visualized. The bands are labeled according to expected relative migration pattern, and VP2 is identified by its absence in the 75S peak. The ratio of VP0 to VP3 bands was analyzed from four independent experiments and plotted in arbitrary units. * p<0.05, ** p<0.01, *** p<0.0001.

We wanted to determine which of these post-RNA replication steps is promoted by vesicle acidification. To analyze virions, we infected cells at an MOI of 50 pfu/cell and labeled viral proteins with ^35^S-Methionine beginning at 3 h.p.i. At 5 or 6 h.p.i., cells were collected and gently lysed, and lysates were separated on a continuous 15–30% sucrose gradient. Fractions were collected and analyzed for radioactive content. **Figure 6A** shows the ^35^S counts per minute (CPM) of fractions from cultures infected in the presence or absence of NH_4_Cl. In each case, we observe the two expected peaks, 150S and 75S. The 75S peak consists of empty capsids, while the 150S peak fractions contain encapsidated genomes, only some of which are mature and infectious. We found no significant change in the size or magnitude of the 150S peak, although there was a small but consistent shift in the NH_4_Cl-treated samples, with both peaks having slightly higher mobility in the gradient. At 5 h.p.i. we observe essentially no change in the peaks resulting from NH_4_Cl treatment. At 6 h.p.i., we observe an increase in the magnitude of the 75S peak, but little change in the 150S peak, which contains the infectious virions. We conclude that the loss of pfu resulting from NH_4_Cl treatment is likely not due to defects in particle assembly genome incorporation, as such defects would likely result in a reduction in the amount of 150S particles.

To confirm that NH_4_Cl was having the expected effect on viral titer, we measured pfu from the pooled 150S peak fractions. (**Figure 6B**) As in previous experiments, pfu was reduced by about one log in the presence of NH_4_Cl. Since the size of the peak was not altered by NH_4_Cl treatment, we hypothesized that the maturation of 150S particles is promoted by vesicle acidification. We pooled the three 150S and three 75S peak fractions and ran them on an SDS-PAGE. The 75S peak should contain exclusively VP0, VP1, and VP3, whereas in the 150S peak VP0 levels will be depleted and replaced with the cleavage products VP2 and VP4 as provirions mature to infectious virions. For reasons that are unclear, labeled VP4 is difficult to detect by SDS-PAGE [56]–[59]. In **Figure 6C** we identify three labeled bands in 75S fractions in both treated and untreated cells, which we have labeled as VP0, VP1, and VP3 based on classical relative molecular weight observations [55], [57]. In the 150S fraction, we identify the VP2 band at its expected migration between VP1 and VP3. In the presence of NH_4_Cl, the VP2 band is reduced and the VP0 band is more easily detected. We have quantitated these bands from four independent experiments and graphed the ratio of VP0 protein to the levels of VP3, which is not cleaved during maturation. We find that NH_4_Cl treatment significantly increases the amount of VP0 present, indicating that vesicle acidification promotes the maturation of encapsidated virus particles into mature, infectious virions.

**Figure 7:**
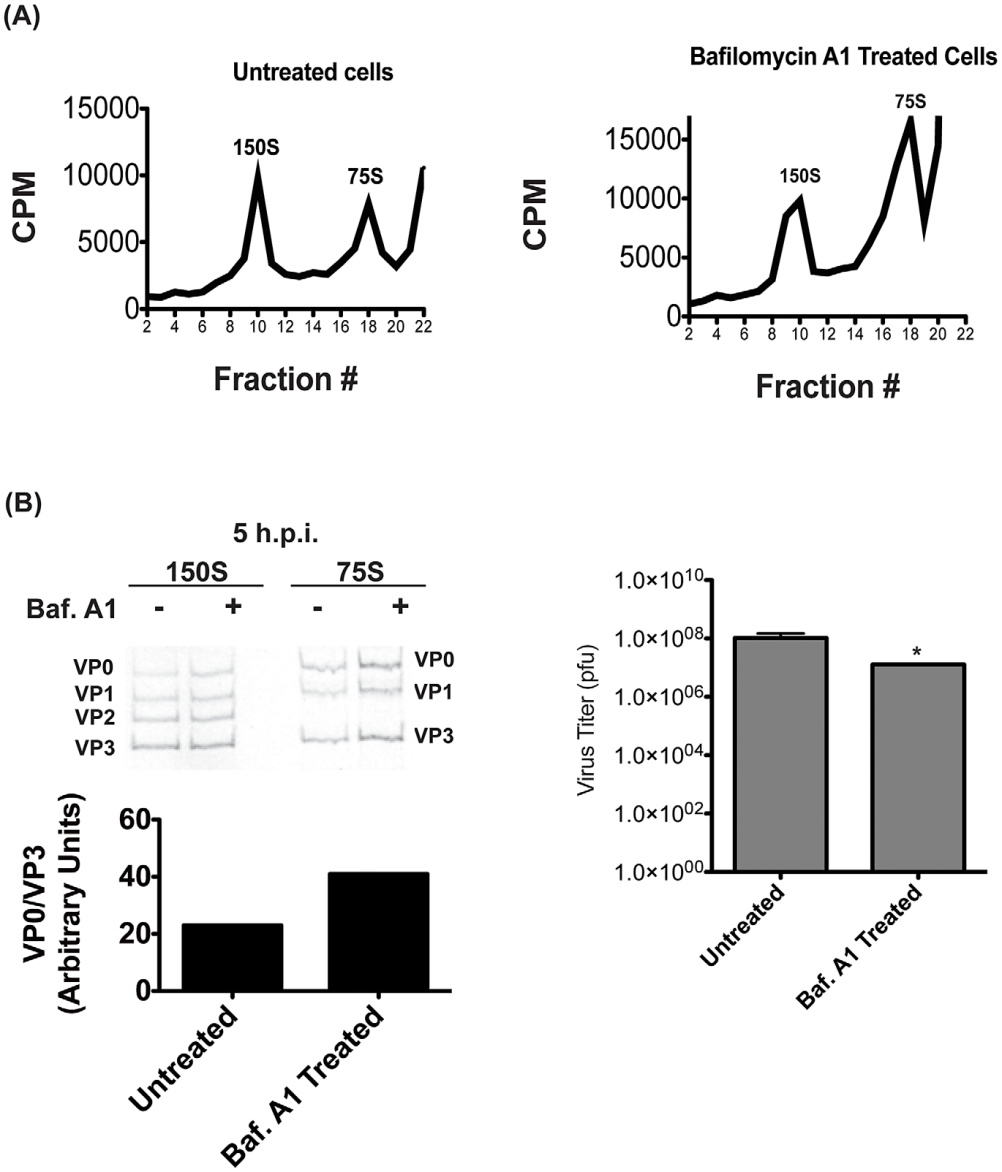
Proton pump inhibition also affects virion maturation. (A) H1-Hela cells were infected at an MOI of 50 pfu/cell, and half of the samples were treated with bafilomycin A1 as described in Figure 4. Cells were labeled with ^35^S-Methionine from 3 h.p.i. until collection at 5 h.p.i., and lysates were then separated on a 15–30% sucrose gradients. Fractions were then collected and the counts per minute (CPM) were analyzed for each fraction. (B) Three fractions representing the 150S and 75S peaks were pooled and run on SDS-PAGE as in Figure 6. The ratio of VP0 to VP3 bands was analyzed and plotted in arbitrary units. The titer of the infectious virus in the pooled 150S fractions was determined by plaque assay. * p<0.05.

To confirm that the results were not specific to NH_4_Cl treatment, we repeated these sucrose gradient purification experiments using bafilomycin A1 to inhibit vesicle acidification. We again see no change in the 150S peak containing encapsidated genomes, although the 75S peak appeared to increase in size. (**Figure 7A**) As with NH_4_Cl treatment, we found an increase in VP0 abundance when vesicle acidification is inhibited, indicating that VP0 cleavage is inhibited. (**Figure 7B**) These data are consistent with the data in Figure 6, and indicate that NH_4_Cl is having the expected effect on vesicle acidification. Taken together, our data show that autophagosome formation promotes viral RNA replication, while vesicle acidification promotes maturation of the assembled, encapsidated virus particles into infectious virions.

## Discussion

Many pathogens that induce autophagic signaling to promote their own replication also inhibit autophagic degradation, presumably to prevent their own destruction by the autophagy machinery. This idea is based on experiments using autophagy-subverting pathogens such as Coxsackievirus and *Legionella pneumophila* [20], [21], [60]. In contrast, we have shown that poliovirus, a pathogen whose replication is promoted by autophagic signaling, induces *bona fide* autophagic protein degradation. However, autophagic degradation does not itself promote poliovirus production. Instead, we find that acidification of cellular compartments is required for normal virus production. Specifically, we have shown that inhibitors of acidification inhibit maturation of virus particles into infectious virions. However, we do not know which cellular compartment is acidifying to promote PV maturation. Since cellular autophagosomes promote PV replication and become acidic prior to their fusion with the lysosome, autophagosomes are an attractive candidate for an acidic compartment to promote virion maturation [18].

During poliovirus infection, autophagic signaling can be identified by 3 h.p.i., and by 5 h.p.i. the cytoplasm is filled with double-membraned vesicles [15], [32], [47]. These autophagosome-like objects, abundant late in infection, appear to be full of viral particles, a phenomenon which has never been explained [2]. Virus-induced autophagosome-like vesicles are marked with proteins from the viral RNA replication complex, and poliovirus, like all positive-strand RNA viruses, replicates its RNA in association with cellular membranes [12], [15]. These data have lead to the hypothesis that autophagic vesicles are the sites of viral RNA replication. An alternative hypothesis for the site of RNA replication suggests that vesicles derived from the secretory pathway provide a substrate for replication complexes [61], [62]. For Coxsackievirus B3, the reorganization of secretory vesicles generates a novel membrane structure to act as a substrate for viral RNA replication complexes [63]. However, since CVB3 does not induce autophagic degradation, there may be important differences between the PV and CVB3 structures [20], [21].

**Figure 8:**
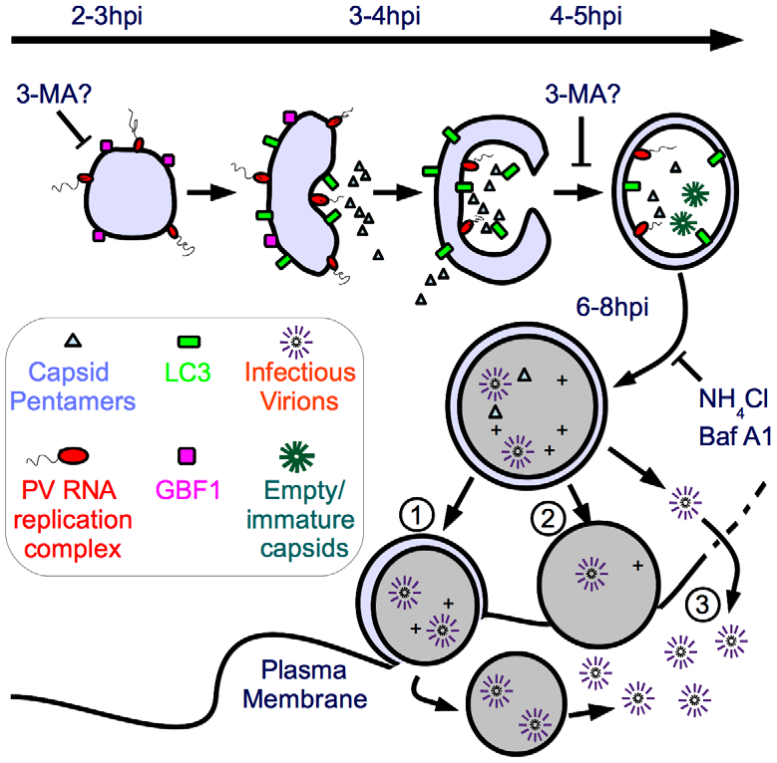
Model of potential roles of autophagosome-amphisome in PV production and cell exit. We have demonstrated that initiation of autophagy is required for viral RNA replication, while vesicle acidification is required for maturation of virus particles. In our model, by 2–3 h post-infection processed 3A protein integrates into single-membraned structures resembling secretory vesicles, which become the initial sites of viral RNA replication. 3-MA either inhibits formation of these single-membraned vesicles which act as the site of RNA replication, or inhibits the progress of these vesicles into double-membraned vesicles, which act as replication sites. As the peak of viral genome replication (3 h) passes, the autophagic machinery is engaged to change the single-membraned vesicles into double-membraned vesicles. As a result, some of RNA replication complexes and capsid proteins are trapped within the double-membraned vesicles, while some are cytosolic. As the amphisomes acidify, particle maturation is more efficient inside the vesicles. Maturation of the amphisomes can be inhibited using NH_4_Cl or bafilomycin A1. The mature virions, surrounded by two membranes, have a topological problem in exiting the cell. We propose three possible mechanisms for exit. [**1**] The outer membrane fuses with the plasma membrane, releasing a short-lived labile single-membraned vesicle containing virus, which eventually is released into the extracellular milieu. [**2**] The double-membraned vesicles lose one membrane, as occurs during maturation of cellular autophagosomes, and the single-membraned vesicles fuse with the plasma membrane. [**3**] Virus exits the vesicle into the cytoplasm due to breakup of the vesicle or another mechanism. Virus then exits the cell when lysis occurs at approximately 8 h post-infection.

Recently, EM tomography carried out on PV-infected cells fixed at different h.p.i. suggested that single-membraned vesicles, seen early in infected cells, may be connected to the double-membraned vesicles seen later in infected cells [64]. Research in the autophagy field suggests that vesicles of the secretory pathway are used in the generation of autophagosomes [65], [66]. Therefore, it is possible that the two hypotheses for the origin of viral replication vesicles are both correct. We propose such a “unified model”, in which single-membraned secretory-derived vesicles predominate at the peak of RNA replication (2.5 h.p.i.–3.5 h.p.i.) and act as the substrate for RNA replication complexes. (**Figure 8**) Then, using the cell's autophagic machinery, these vesicles morph into the double-membraned vesicles observed later during infection (4 h.p.i.–6 h.p.i.).

If this model is correct, then it is likely that during the change from single-membraned vesicles to double-membraned vesicles, a significant amount of newly synthesized viral RNA and viral proteins would be taken up into the lumen of the autophagosome-like structures. This would explain the observation of virus particles inside autophagosomes. If the virus only needs the cytosolic face of membranes as a substrate for RNA replication, then the effect of acidification inhibitors would not be significant. However, if characteristics unique to the environment within the vesicle, such as low pH, are important for virus production, then acidification inhibitors would be expected to reduce viral yield. We have provided the first experimental evidence to support this second scenario.

Our data demonstrate that inhibition of autophagosome formation by 3-MA affects poliovirus RNA replication. In the context of our model, there are two possible roles for 3-MA. (**Figure 8**) One, it could inhibit production of single-membraned secretory pathway-derived vesicles, which represent an early step in formation of the autophagosome. This makes sense in context of recent data showing single-membraned vesicles physically associating with double-membraned vesicles as infection progresses [64]. Alternatively, 3-MA could allow single-membraned vesicles to form, but inhibit the formation of double-membraned vesicles, which then act as primary sites of RNA replication. Although this is a formal possibility, we think it less likely in the context of the data indicating an important role for secretory-derived vesicles in viral RNA replication [61], [63]. In either case, 3-MA inhibits viral RNA replication and all downstream steps in virus production. NH_4_Cl, however, allows production of early autophagic vesicles, inhibiting only their acidification and maturation, which specifically reduces VP0 cleavage. Inhibiting acidification has no effect on viral genomic RNA replication. These data make it clear that vesicle acidification is important for maturation of newly-assembled poliovirus provirions into infectious particles.

There are several possibilities to explain why vesicle acidification promotes virus maturation. It is important to note that because we do not have the technical ability to inhibit acidification of specific compartments, it is possible that acidification of another cellular compartment, not amphisomes, is key to virus production. It is also possible that treating cells with bafilomycin A1 or NH_4_Cl alters organelle structure or membrane trafficking in a way that inhibits virus production. We think these explanations are unlikely due to the important role autophagosomes play in virus production and the observed presence of virus particles inside autophagosomes [2]. A second possibility is that autophagosomes act as “sponges” for ions, increasing the pH of the cytosol to promote virion maturation [67]. We believe this is unlikely because PV is an enteric virus, capable of remaining infectious in the low pH environment of the human gut, and should not be adversely affected by low pH.

Our favored hypothesis is that virions taken up inside acidic amphisomes have a greater likelihood of maturing into infectious virions. (**Figure 8**) Several pieces of data lead us to believe that the amphisome interior a likely site of virus maturation. First, autophagosome formation is required for normal viral RNA replication, indicating that newly synthesized viral genomes are likely to be associated with, or in close proximity to, autophagosomes. Second, recent published evidence showed that PV-induced double-membraned vesicles are derived from RNA replication-associated vesicles. Finally, PV-induced amphisomes, which stain with the viral RNA replication protein 3A, are clearly acidic due to their co-staining with the lysosomotropic agent monodansylcadaverine [12].

The majority of virus particles observed during infection are cytosolic, which appears to pose a problem. How can an important function of virus maturation occur in a compartment containing a small percentage of particles? First, the particle-to-pfu ratio of PV has been measured as anywhere from 30 to 1000 [68], [69]. This means that even at the lowest estimate, the vast majority of particles produced during an replication cycle are not infectious. We find that inhibiting acidification eliminates about 90% of pfu. (**Figure 4**) Therefore, it is not unreasonable to consider that the particles inside amphisomes may have a much better chance of maturing into infectious virus than those outside the amphisome. In our hands, 10% of pfu are resistant to treatment with acidification inhibitors, and we see evidence of residual VP0 cleavage in the presence of NH_4_Cl or bafilomycin A1. (**Figures 6** **,** **7**) This could be for multiple reasons. First, our treatments do not completely block either autophagy or vesicle acidification. Second, there may certainly be residual cleavage of VP0 in the cytosol or at neutral pH. In any case, our data do not show that *only* particles inside acidic compartments will mature.

Our model is that poliovirus particle maturation is more efficient for particles within amphisomes. This study therefore has important implications for our understanding of the PV life cycle, because all steps in virus production have long been thought to occur in the cytoplasm. There are few reports in the literature showing that any stage in positive-strand virus production is not cytoplasmic. Picornavirus particles have been observed in autophagosomes, providing a precedent for our hypothesis [2], [70]. In addition, the alphavirus Brome Mosaic Virus (BMV) replicates its RNA within invaginations of the endoplasmic reticulum, and the passage of macromolecules through the neck of these compartments is believed to be tightly regulated by the virus [71]. However, it is clear from the data presented here that the compartmental requirement for PV is very different from BMV. Normal levels of poliovirus production require vesicle acidification for a post-RNA replication step or steps. (**Figures 4** **–** **7**)

Virus particles are not truly infectious until the mature particles exit the cell. Viral egress is a poorly understood process, often proposed to be the result of the cytopathic effect at the end of an infectious cycle [72]–[74] Previously it was found that the effect of modulating autophagy is greater on pre-lytic exit of infectious virus than on cell-associated virus [12], [75] Our data are consistent with the model that a higher percentage of packaged, infectious virus assembled and released as a result of increased autophagy.

Vesicles can be induced by the transfection of viral replication proteins into cells, but when those cells are superinfected with PV, the pre-formed vesicles are not used for viral RNA synthesis [76]. These data point to a coupling between RNA replication and vesicle generation and may indicate a viral strategy to ensure that at least some newly replicated RNA reaches the luminal side of the vesicles. If our model is correct and PV particles inside autophagosomes are more likely to be mature, infectious virus, then the viruses inside these double-membraned structures need a mechanism to exit the cell. It is also possible that the steps of virus assembly and encapsidation may occur in the cytosol, but maturation occurs inside acidic vesicles. However, we find this unlikely, as immature virions would face a topology problem, having to cross two membranes to enter the amphisome for efficient maturation cleavage. Therefore, we propose that it is most likely that virions are being assembled and encapsidated inside vesicles.

As a non-enveloped virus, poliovirus is not thought to be found in the wild surrounded by a lipid envelope. Therefore, if poliovirus genome encapsidation occurs in autophagosomes, then there must be some mechanism by which virus can exit from these double-membraned vesicles. We describe some possibilities in **Figure 8**. The simplest model is that the autophagosome fuses with the plasma membrane, releasing virus into the extracellular space. There are data for such a model in poliovirus, and more recent data has confirmed the existence of a minor secretory pathway linked to autophagy [77]–[80]. However, this fusion would likely have to occur after the double-membraned amphisome is converted to a single-membraned autolysosome, because fusion of a double-membraned vesicle with the plasma membrane would release a cytosol-filled single-membraned vesicle into the extracellular space. In the case of infection, this would release a vesicle presumably filled with multiple virions. Poliovirus is not found in extracellular vesicles, so any such structures would presumably be very short-lived and difficult to detect. Alternatively, there could be a mechanism by which virions re-enter the cytosol after the pH-dependent step. These cytoplasmic viruses would then be released by cell lysis.

Our data indicate that acidic amphisomes promote the late, post-RNA replication step of poliovirus particle maturation. The idea that the interior of these vesicles may be the site of virion assembly, genome packaging, maturation, and cell egress has the potential to alter the models in the field describing the latter part of picornavirus infection.

## Supporting Information

Figure S1 **Effects of 3-MA treatment of H1-HeLa and 293T cells.** (**A**) Cells were either treated with 20 mM 3-MA as described in Figure 5 or untreated, then infected with PV at an MOI of 50 pfu/cell or mock infected. Intracellular virus was collected and titered by plaque assay. (**B**) Lysates from parallel infections to (A) were run on SDS-PAGE and blots probed for LC3 and GAPDH. (**C**) Triplicate plates of H1-Hela cells were infected at an MOI of 0.1, and 3-MA was added to the media at the time of infection. Total RNA was harvested at 6 h.p.i., and the levels of viral genomic RNA and host GAPDH RNA were measured by qRT-PCR. Viral RNA was normalized to GAPDH levels using the delta Ct method. Guanidine-HCl treatment was performed as described in Figure 5, and data were pooled from three replicate experiments. The titer of intracellular virus from each replicate at 6 h.p.i. was determined by plaque assay. (**D**) H1-Hela cells were infected at an MOI of 50 pfu/cell, and half of the samples were treated with 20 mM 3-MA. Cells were labeled with ^35^S-Methionine from 3 h.p.i. until collection at 6 h.p.i., and lysates were then separated on a 15–30% sucrose gradients. Fractions were then collected and the counts per minute (CPM) were analyzed for each fraction. Representative gradients from three independent experiments are shown. (**E**) Three fractions representing the 150S and 75S peaks were pooled and run on SDS-PAGE, and the ^35^S-Methionine labeled bands were visualized. The bands are labeled according to expected relative migration pattern, and VP2 is identified by its absence in the 75S peak. The three fractions representing the 150S peak in each experiment were then pooled for plaque assay analysis. ** p<0.01, *** p<0.0001.
(TIF)
