# Supplementary figures and images for: Intracellular Vesicle Acidification Promotes Maturation of Infectious Poliovirus Particles
Source: PLoS Pathog. 2012 Nov 29;8(11):e1003046. doi: 10.1371/journal.ppat.1003046 (PMC3510256; doi:10.1371/journal.ppat.1003046)

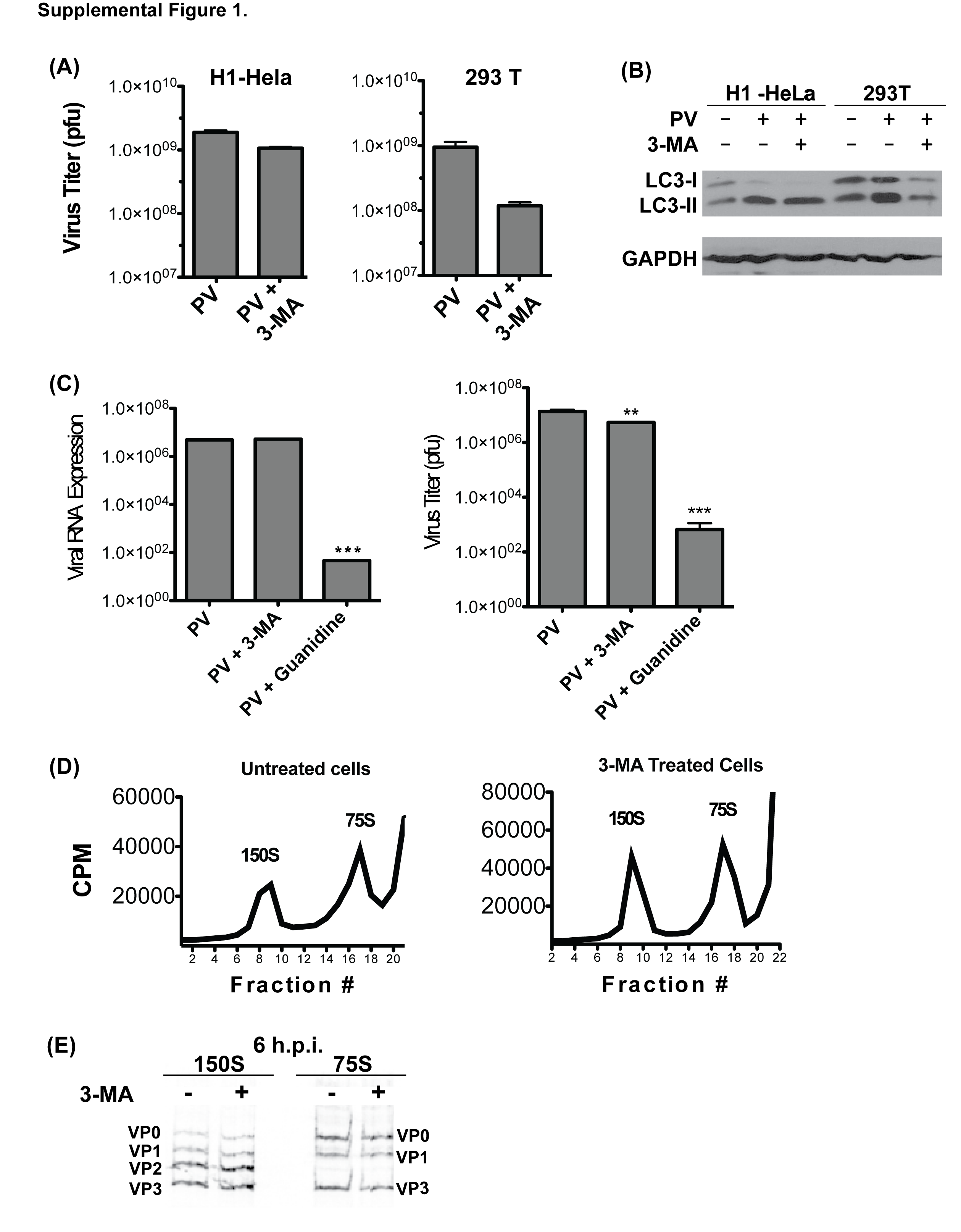

Supplement: Figure S1 — Effects of 3-MA treatment of H1-HeLa and 293T cells. (A) Cells were either treated with 20 mM 3-MA as described in Figure 5 or untreated, then infected with PV at an MOI of 50 pfu/cell or mock infected. Intracellular virus was collected and titered by plaque assay. (B) Lysates from parallel infections to (A) were run on SDS-PAGE and blots probed for LC3 and GAPDH. (C) Triplicate plates of H1-Hela cells were infected at an MOI of 0.1, and 3-MA was added to the media at the time of infection. Total RNA was harvested at 6 h.p.i., and the levels of viral genomic RNA and host GAPDH RNA were measured by qRT-PCR. Viral RNA was normalized to GAPDH levels using the delta Ct method. Guanidine-HCl treatment was performed as described in Figure 5, and data were pooled from three replicate experiments. The titer of intracellular virus from each replicate at 6 h.p.i. was determined by plaque assay. (D) H1-Hela cells were infected at an MOI of 50 pfu/cell, and half of the samples were treated with 20 mM 3-MA. Cells were labeled with 35S-Methionine from 3 h.p.i. until collection at 6 h.p.i., and lysates were then separated on a 15–30% sucrose gradients. Fractions were then collected and the counts per minute (CPM) were analyzed for each fraction. Representative gradients from three independent experiments are shown. (E) Three fractions representing the 150S and 75S peaks were pooled and run on SDS-PAGE, and the 35S-Methionine labeled bands were visualized. The bands are labeled according to expected relative migration pattern, and VP2 is identified by its absence in the 75S peak. The three fractions representing the 150S peak in each experiment were then pooled for plaque assay analysis. ** p<0.01, *** p<0.0001. (TIF) [file ppat.1003046.s001.tif]
